# Concave Pt–Zn Nanocubes with High‐Index Faceted Pt Skin as Highly Efficient Oxygen Reduction Catalyst

**DOI:** 10.1002/advs.202200147

**Published:** 2022-02-24

**Authors:** Mengli Liu, Bang‐An Lu, Gege Yang, Pengfei Yuan, Huicong Xia, Yajin Wang, Kai Guo, Shuyan Zhao, Jia Liu, Yue Yu, Wenfu Yan, Chung‐Li Dong, Jia‐Nan Zhang, Shichun Mu

**Affiliations:** ^1^ College of Materials Science and Engineering Zhengzhou University Zhengzhou 450000 P. R. China; ^2^ International Joint Research Laboratory for Quantum Functional Materials of Henan Province and School of Physics and Microelectronics Zhengzhou University Zhengzhou 450000 P. R. China; ^3^ Shanghai Hydrogen Propulsion Technology Co., Ltd. Shanghai 200000 P. R. China; ^4^ State Key Laboratory of Inorganic Synthesis & Preparative Chemistry Jilin University Changchun 130000 P. R. China; ^5^ Department of Physics Tamkang University New Taipei City Taiwan; ^6^ State Key Laboratory of Advanced Technology for Materials Synthesis and Processing Wuhan University of Technology Wuhan 430070 P. R. China

**Keywords:** high‐index facets, oxygen reduction reaction, Pt–Zn catalyst, ultrathin Pt skin

## Abstract

High dosage of expensive Pt to catalyze the sluggish oxygen reduction reaction (ORR) on the cathode severely impedes the commercialization of proton exchange membrane fuel cells. Therefore, it is urgent to cut down the Pt catalyst by efficiently improving the ORR activity while maintaining high durability. Herein, magic concave Pt–Zn nanocubes with high‐index faceted Pt skin (Pt_78_Zn_22_) are proposed for high‐efficiency catalysis toward proton exchange membrane fuel cells. These unique structural features endow the Pt‐skin Pt_78_Zn_22_/KB with a mass activity of 1.18 mA μg_Pt_^–1^ and a specific activity of 3.64 mA cm^–2^ for the ORR at 0.9 V (vs RHE). Meanwhile, the H_2_–O_2_ fuel cell assembled by this catalyst delivers an ultrahigh peak power density of ≈1449 mW cm^–2^. Both experiments and theoretical calculations show that the electronic structure of the surface is adjusted, thereby shortening the length of the Pt–Pt bond and reducing the adsorption energy of OH*/O* on the Pt surface. This work demonstrates the synergistic effect of the oxidation‐resistant metal Zn and the construction of Pt‐rich surface engineering. Also, it guides the future development of catalysts for their practical applications in energy conversion technologies and beyond.

## Graphical abstract

Such excellent performance of the Pt_78_Zn_22_/KB catalyst can be attributed to the incorporation of zinc and the existence of high‐index facets ultrathin Pt‐rich skins, which adjust the electronic structure of Pt on the surface, and leads to improved catalytic activity. This work provides a feasible strategy for designing Pt–M electrocatalysts and helps the energy conversion technology be applied in practice.

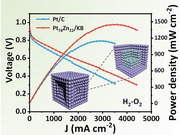

## 1 Introduction

The proton exchange membrane fuel cells (PEMFCs) are the well‐known attractive alternative to fossil fuel combustion with high energy efficiency.^[^ ^1^, ^2^, ^3^, ^4^ ^]^ However, their commercialization is significantly impeded by the sluggish kinetics of the cathodic oxygen reduction reaction (ORR) and considerable amounts of platinum (Pt)‐based materials as the most active and stable catalyst towards ORR.^[^ ^5^, ^6^, ^7^, ^8^, ^9^, ^10^, ^11^ ^]^ In the past, Pt‐based catalysts integrated with transition metals (Pt–M), especially with 3d transition metals (Fe, Ni, Co, Cu, etc.),^[^ ^12^, ^13^, ^14^, ^15^, ^16^ ^]^ have been extensively developed to reduce Pt loading by increasing the activity of Pt. These alloying transition metals would inevitably dissolve during the practical operation of PEMFCs, which leads to the combination of metal ions and H_2_O_2_ generated during the reaction, thereby degrading the proton exchange membrane by a Fenton reaction producing aggressive ⋅OH radical. Moreover, the resulting metal ions would decrease the conductivity of the proton‐exchange membrane.^[^ ^17^, ^18^, ^19^, ^20^ ^]^ Among these transition metal elements, Zn exhibits better antioxidation properties, which can effectively inhibit Fenton reaction, thereby enhancing the stability of Pt‐based catalysts.^[^ ^21^, ^22^, ^23^ ^]^

Recently, considerable efforts have been focused on tuning the structure, composition, and morphology.^[^ ^24^, ^25^, ^26^, ^27^, ^28^, ^29^, ^30^, ^31^, ^32^, ^33^ ^]^ As we all know, the catalytic reaction generally occurs on the surface of catalysts, and thus the surface structures play a vital role in determining performance.^[^ ^34^, ^35^ ^]^ Previous results also give a clear picture of ORR structure sensitivity and reveal that stepped surfaces are more active than low index planes due to the surface‐dependent adsorption behavior of spectator species.^[^ ^36^, ^37^ ^]^ Another important strategy to enhance the catalytic activity is customizing the metal nanocrystals with high‐index facets. The atoms located at steps, ledges, and kinks of high‐indexed nanocrystals can act as additional catalytically active sites,^[^ ^38^, ^39^ ^]^ and are significant to rationally design and prepare shape‐controlled nanocatalysts with high density of low‐coordinated atoms, beneficial to catalysis.^[^ ^40^ ^]^ However, up to now, there is almost no clear design with ultrathin Pt skin and high index faceted synergistic Pt–Zn‐based catalysts for efficient ORR. Therefore, the combination strategy of Pt‐skin and high‐index structural strategies were selected aiming at the high ORR performance in this work because there is still a great challenge in developing highly active and inexpensive Pt–M based catalysts.

In this work, concave Pt–Zn nanocubes with high‐index faceted Pt skin are prepared by a facile solvothermal method. The formation of this high‐index Pt–Zn catalyst is based on the reduction ability of aldehyde groups in solvothermal reactions. The concave nanocube shape provides a more active surface structure, such as atomic steps and edges, and also improves the utilization of Pt. The Pt_78_Zn_22_/KB shows prominent acid ORR activity and robust stability as expected. The origin of such outstanding performance was in‐depth investigated through in situ synchrotron radiation X‐ray absorption fine structure (XAFS) analysis and X‐ray photoelectron spectroscopy (XPS) analysis. DFT calculations show that the adsorption energy of OH*/O* on the surface of a high‐index faceted model with ultrathin Pt skin is weaker than that of Pt (111). These findings demonstrate that these new classes of high‐index faceted Pt–Zn nanostructures are promising material candidates with much‐enhanced performance for practical PEMFCs applications.

## 2 Results and Discussion

**Scheme 1:**
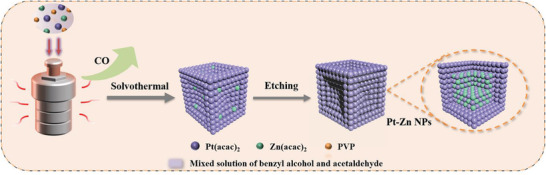
Schematic illustration of the preparation of the Pt–Zn/KB catalyst.

A facile one‐pot approach fabricated the concave Pt–Zn nanocrystals (NCs) with high‐index faceted ultrathin Pt skin (**Scheme** **1**). Briefly, platinum acetylacetonate [Pt(acac)_2_], zinc acetylacetonate [Zn(acac)_2_], polyvinyl pyrrolidone (PVP), benzyl alcohol, and acetaldehyde mixtures were stirred vigorously and then heated at 180 ℃ for 8 h. Subsequently, Pt–Zn/Ketjen Black (Pt–Zn/KB) NCs were immersed in the 1 m acetic acid (60 ℃) solution for 4 h to remove Zn atom on the surface to obtain concave Pt–Zn nanocrystals (NCs) with high‐index faceted ultrathin Pt skin samples. In the synthesis process of concave nanocubes, PVP and benzyl alcohol were used as surfactants and solvents, respectively, while acetaldehyde was a reducing agent. We carried out a series of experiments to explore the origin of the formation of concave surface. To better understand the formation process of concave nanocube, the intermediate nanocrystals produced by adding different amounts of acetaldehyde were studied by transmission electron microscopy (TEM). Figures S1–S3 (Supporting Information) detail the morphological evolution of the Pt–Zn nanocrystals with increasing acetaldehyde content. In the absence of acetaldehyde, only irregular nanoparticles can be obtained. With the increase of amounts of acetaldehyde, nanocubes can be synthesized, and concave nanocubes can be fabricated when 6 mL acetaldehyde is introduced (Figure S4, Supporting Information). As suggested by previous work, CO generated by the decomposition of aldehydes can prefer adsorption on {100} terraces and promote the formation of concave structure. ^[^ ^41^ ^]^ Using the same preparation procedure, by adjusting the molar ratio of Pt/Zn, Pt_46_Zn_54_/KB, Pt_78_Zn_22_/KB, and pure Pt/KB were prepared, respectively. The weight percent of Pt and Zn are analyzed by the inductively coupled plasma‐optical emission spectrometer (ICP‐OES) analysis. As listed in Table S1, the weight percent of Pt is 24.02% for Pt_78_Zn_22_/KB, 13.1% for Pt_46_Zn_54_/KB, 25.75% for pure Pt/KB, respectively. More interesting, the atomic ratios of metallic precursors also play a vital role in forming concave nanocubes, indicating that the underpotential deposition of Zn onto Pt may participate in the formation of concave structures.^[^ ^42^, ^43^ ^]^

**Figure 1:**
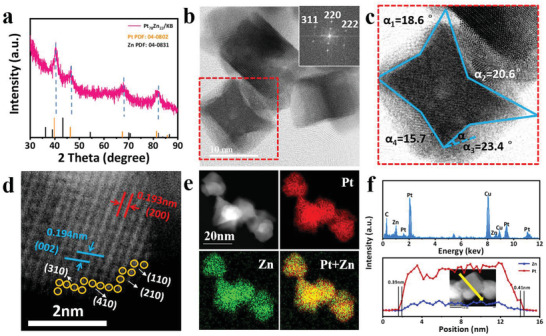
Structure and morphology. a) XRD patterns of Pt_78_Zn_22_/KB. b) HAADF‐STEM image of the as‐prepared concave Pt_78_Zn_22_ NCs (inset is the corresponding FFT pattern). c) Enlarge HAADF‐STEM image of the red area indicated in (b). d) HAADF‐STEM image of Pt_78_Zn_22_. The red and blue dots and arrows indicate the lattice spacing. e) HAADF‐STEM image and elemental mappings of Pt, Zn and overlapping in Pt_78_Zn_22_. f) EDS profile of Pt_78_Zn_22_ /KB (top) and STEM‐EDS line‐scanning profile (bottom) of a single nanoparticle. The inset shows the studied nanoparticle and the line‐scanning analysis.

**Figure** **1**a and Figure S5 (Supporting Information) show the powder X‐ray diffraction (PXRD) patterns of Pt_78_Zn_22_/KB NCs, pure Pt/KB, and Pt_46_Zn_54_ /KB. The diffraction peaks of Pt_78_Zn_22_/KB NCs and Pt_46_Zn_54_ /KB show a slight positive shift relative to Pt (JCPDS No. 04‐0802), indicating the incorporation of Zn atom into the lattice of Pt. The high angle annular dark field‐scanning transmission electron microscopy (HAADF‐STEM) images emphasize the uniform morphology of a Pt_78_Zn_22_ with a length of ≈10 nm (Figure 1b and Figure S4, Supporting Information). Meanwhile, samples without Zn and another ratio of Pt/Zn were also synthesized for comparison and denoted as “pure Pt/KB” and “Pt_46_Zn_54_/KB,” respectively. Both Pt_46_Zn_54_ and pure Pt are nanoparticles with an average particle size of ≈4.9 nm (Figures S6 and S7, Supporting Information). Figure 1c shows an enlarged HAADF‐STEM image of an individual Pt_78_Zn_22_ NCs concave nanocube to determine the surface facets of the nanocrystal. The Miller indices can be derived from the projection angles along the selected crystal axis for a concave nanocube surrounded by high‐index facets.^[^ ^44^, ^45^ ^]^ The angles were measured in the range from 15.7° to 23.4°. In comparison with the theoretical angles of the different faces {*hk*0} listed in Table S2 (Supporting Information), the corresponding surface faces vary from {310} to {830}.^[^ ^46^ ^]^ The inset of Figure 1b shows the single‐crystalline structure of the concave Pt_78_Zn_22_ nanocube by Fourier transform (FT) pattern. The two different lattice fringes revealed by the HAADF‐STEM image are 0.193 and 0.194 nm, which correspond to crystal planes (200) and (002), respectively (Figure 1d). In addition, the high‐index facets of {210}, {310}, {410} can be readily observed, which would possess a higher density of low‐coordinated sites compared to low‐indexed surface.^[^ ^39^ ^]^ According to the Z‐contrast, it can be concluded that the doping of zinc atoms replaces part of the platinum atoms, resulting in a smaller lattice fringe spacing. Energy‐dispersive X‐ray spectroscopy (EDS) was further employed to explore the composition of NCs. As shown in Figure 1e, the EDS elemental mapping profiles demonstrate the distributions of Pt and Zn. Besides, the recorded profile along the yellow arrow of the NCs further confirms the coexistence of the Pt and Zn in the NCs (Figure 1f). The inset shows the EDS line scanning of a single nanoparticle in Pt_78_Zn_22_/KB, confirming a well‐defined Pt‐skin structure of the NCs formed. The thickness of the outer shell along the direction of the yellow arrow was 0.39 and 0.41 nm, respectively, corresponding to approximately two atomic Pt layers.

**Figure 2:**
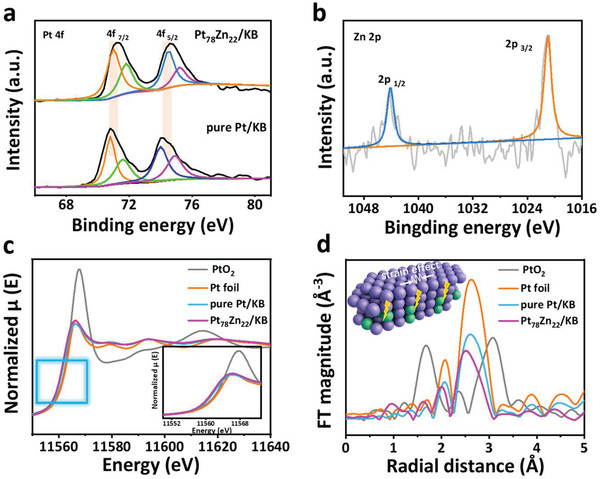
XPS and XAS analysis of the catalysts. a) Pt 4f XPS spectra of Pt_78_Zn_22_/KB, and pure Pt/KB. b) Zn 2p XPS spectra of Pt_78_Zn_22_/KB. c) Pt L_3_‐edge XANES and with an enlarged view of the area marked by the blue square. d) Fourier‐Transform EXAFS spectra of Pt_78_Zn_22_/KB and reference samples.

X‐ray photoelectron spectrum (XPS) was used to identify the distribution and chemical state of Pt_78_Zn_22_/KB NCs and pure Pt/KB NCs. The Pt 4f_7/2_ binding energy of Pt_78_Zn_22_/KB (71.0 eV) positively shifts 0.2 eV relative to pure Pt/KB (**Figure** **2**a), the shift of Pt bonding energy suggests that the electronic properties of Pt in Pt_78_Zn_22_/KB are tuned due to the alloying with elemental Zn.^[^ ^47^, ^48^ ^]^ The electron transfer from Zn to Pt leads to the upshift of reference level as well as the downshift of d band center, and thus the positive shift of Pt 4f_7/2_ core level observed in XPS measurements.^[^ ^49^ ^]^ The downshift of the d‐band center can weaken the adsorption energy of oxygenate intermediates and facilitate the kinetics of oxygen reduction.^[^ ^50^, ^51^, ^52^ ^]^ Moreover, the DFT calculation of the projection electron density of states (PDOS) shows that the d‐band centers of pure Pt (111) and Pt_78_Zn_22_ (210) are −1.86 and −2.128 eV, respectively (Figure S8, Supporting Information). The d‐band center of Pt_78_Zn_22_ (210) has a significant negative shift relative to pure Pt, consistent with the analysis of XPS. In the Zn 2p XPS spectra of Pt_78_Zn_22_/KB, Zn 2p_3/2_ at 1021 eV has a negative shift related to Zn metal (1021.7 eV) (Figure 2b), corresponding to the electron transfer from Pt to Zn. Besides, no surface ZnO species can be observed.^[^ ^53^ ^]^ Importantly, we used X‐ray absorption near‐edge structure (XANES) and extended X‐ray absorption fine structure (EXAFS) measurements to probe the local metal‐site coordination environment for Pt and Zn.^[^ ^54^ ^]^ Figure 2c shows the Pt L_3_‐edge EANES of Pt_78_Zn_22_/KB, pure Pt/KB, and reference samples. The threshold energy (*E*_0_) of Pt L_3_‐edge on Pt_78_Zn_22_/KB and pure Pt/KB are similar to Pt foil, verifying their metallic state. The white line intensity of Pt L_3_‐edge was used as a qualitative indicator of electron vacancies in the 5d orbitals and exhibited the sequence: Pt foil ≤ pure Pt/KB < Pt_78_Zn_22_/KB < PtO_2_.^[^ ^48^ ^]^ The changes in white line intensity could be caused by the interaction of electrons between Pt and Zn, consistent with the XPS results. As shown in Figure 2d, the EXAFS spectra of Pt L_3_‐edge of Pt_78_Zn_22_/KB reveal that the Pt‐Pt and Pt‐O bond length in Pt_78_Zn_22_/KB is shorter than that of Pt foil and PtO_2_, respectively, which may be caused by the compressive strain effect caused by the addition of Zn.^[^ ^55^, ^56^ ^]^

**Figure 3:**
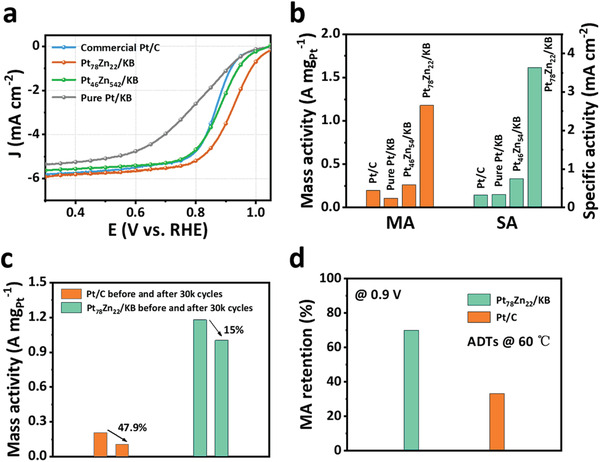
ORR performances of catalysts in 0.1 m HClO_4_. a) Polarization curves of pure Pt/KB, Pt_46_Zn_54_ /KB, Pt_78_Zn_22_ /KB and Pt/C in O_2_‐saturated 0.1 m HClO_4_ at a rotation rate of 1600 rpm (scan rate = 5 mV s^–1^). b) Mass and specific activities pure Pt/KB, Pt_46_Zn_54_/KB, Pt_78_Zn_22_/KB, and commercial Pt/C at 0.9 V. c) The mass activity loss ratio of Pt_78_Zn_22_/KB and commercial Pt/C at 0.9 V after 30k potential cycles. d) Mass activity (MA) retention rate of Pt_78_Zn_22_/KB and commercial Pt/C after 20k ADTs at 0.9 V at 60 ℃.

To evaluate the electrocatalytic ORR activity of samples, the catalysts were deposited on a glassy carbon rotating disk electrode (RDE), and the commercial Pt/C (JM) was used as the benchmark. The cyclic voltammetry (CV) curves of commercial Pt/C, pure Pt/KB, Pt_46_Zn_54_/KB, and Pt_78_Zn_22_/KB electrocatalysts were recorded in Ar‐saturated 0.1 m HClO_4_ aqueous solution (Figure S9, Supporting Information). Considering the adsorption of *H*_upd_ on Pt‐based alloys is highly inhibited, the ECSA obtained by *H*_upd_ is smaller than that of CO stripping. Therefore, we carried out the ECSA measurement of Pt_46_Zn_54_/KB, and Pt_78_Zn_22_/KB based on CO stripping (Figure S10 and Table S3, Supporting Information). The linear scan voltammetry (LSV) in **Figure** **3**a shows that the half‐wave potential of Pt_78_Zn_22_/KB positively shifts by 50 mV relative to the commercial Pt/C catalyst. The mass activity and specific activity of Pt_78_Zn_22_/KB are 1.18 A mg_Pt_^–1^ and 3.64 mA cm^–2^ at 0.9 V versus the reversible hydrogen electrode (RHE), respectively, superior to those of commercial Pt/C (0.205 A mg_Pt_^–1^, 0.325 mA cm^–2^), pure Pt/KB (0.108 A mg_Pt_^–1^, 0.34 mA cm^–2^) and Pt_46_Zn_54_/KB (0.264 A mg_Pt_^–1^, 0.75 mA cm^–2^) catalysts (Figure 3b). The activity of Pt–Zn/KB is more improved than pure Pt/KB, which may be due to the introduction of Zn, but the introduction of excess Zn may reduce the activity. The electron transfer number (*n*) and H_2_O_2_ yield for Pt_78_Zn_22_/KB are about four and less than 5% determined through rotating ring‐disk electrode (Figure S11, Supporting Information), where the direct four‐electron oxygen reduction pathway from O_2_ to H_2_O is confirmed.

More interestingly, Pt_78_Zn_22_/KB NCs also exhibited significantly enhanced stability relative to commercial Pt/C. An accelerated durability test (ADT) with 30 000 potential cycles between 0.6 and 1.0 V at 50 mV s^–1^ in O_2_‐saturated 0.1 m HClO_4_ at room temperature was performed to evaluate the stability. As shown in Figure S12 (Supporting Information), after ADTs, the half‐wave potential negative shift value for Pt_78_Zn_22_/KB is significantly lower than commercial Pt/C (7 mV vs 30 mV). Accordingly, the retained mass and specific activity of Pt_78_Zn_22_/KB were 1.003 mA μg_Pt_^−1^ and 3.47 mA cm^–2^ at 0.9 V, that is, loss of 15% and 5% in mass and specific activity, much lower than 47.9% and 36% of Pt/C (Figure 3c). Also, the loss of the ECSA of Pt_78_Zn_22_/KB is 10% (Figure S13, Supporting Information), also smaller than 20.3% of commercial Pt/C (Figure S14, Supporting Information). Besides, Pt_78_Zn_22_/KB catalyst delivered higher ORR catalytic activity and stability even at an elevated temperature relative to commercial Pt/C catalyst. As shown in Figure S15 (Supporting Information), after 20 000 potential cycles at 60 ℃, the loss of ECSAs for Pt_78_Zn_22_/KB was significantly smaller than commercial Pt/C (12.1% vs 47.2%). Compared to Pt_78_Zn_22_/KB, the commercial Pt/C has undergone more severe degradation of ORR activity (Figure S16, Supporting Information). After ADTs, Pt_78_Zn_22_/KB retains 69.9% and 78.7% of the mass and specific activity, while commercial Pt/C has only 33.3% and 37%, respectively (Figure 3d). However, it should be noted that the two catalysts suffer more severe performance degradation at an elevated temperature relative to room temperature, probably because the high temperature accelerates the corrosion of catalysts. To explore the reasons for the excellent stability of the Pt_78_Zn_22_/KB catalyst, we also performed XPS analysis on the catalyst before and after 10 000 potential cycles to determine whether there is Zn leaching during the electrochemical process. As shown in Figure S17 (Supporting Information), the binding energy of Pt 4f remains unchanged after 10 000 cycles, while the binding energy of Zn 2p has a slight positive shift, indicating that small amounts of Zn are oxidized during ADTs. Figure S18 (Supporting Information) shows the composition provided by XPS. After ADTs, the near‐surface atomic ratio of Zn: Pt ratio decreased from 0.269 to 0.222. Zn atoms appear to be slightly oxidized and leached after 10 000 potential cycles. This stability may be attributed to the construction of Pt skin surface engineering, which inhibit the corrosion and dissolution of Zn atoms from Pt–Zn.^[^ ^57^ ^]^ This method is also suitable for scale‐up production (Figure S19, Supporting Information). The scale‐up production of Pt_78_Zn_22_/KB still has superior performance (*E*_1/2_ = 0.909 V) and can maintain almost the same activity as small batches (1.18 A mg_Pt_^–1^ vs 1.03 A mg_Pt_^–1^). The larger‐scale synthesis is still being explored.

**Figure 4:**
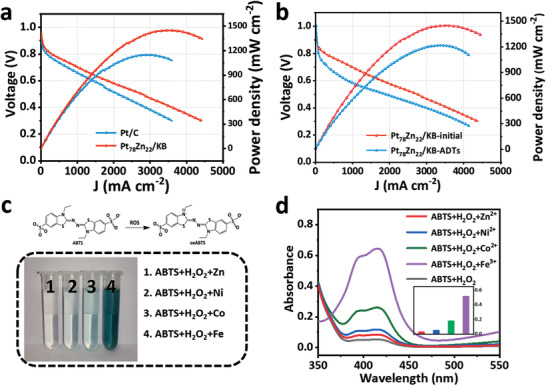
Fuel cell stability performances and anti‐Fenton reaction of catalysts. a) H_2_–O_2_ fuel cell polarization plots with Pt_78_Zn_22_ /KB and Pt/C as the cathode catalysts. b) H_2_–O_2_ fuel cell polarization plots before and after ADTs with Pt_78_Zn_22_/KB as the cathode catalyst. c) Top: Reaction between ROS and ABTS; bottom: photographs showing the color change of the solution containing different metal ions after the Fenton reaction. d) UV/Vis absorption spectra of 0.1 m HClO_4_ solution of ABTS+H_2_O_2_ with Zn, Ni, Co, Fe metal ions and no metal ions. Inset: the absorbance value of the solution at 417 nm. Gray: ABTS+H_2_O_2_; red: ABTS+H_2_O_2_+Zn^2+^; blue: ABTS+H_2_O_2_+Ni^2+^; green: ABTS+H_2_O_2_+Co^2+^; purple: ABTS+H_2_O_2_+Fe^3+^.

Considering the high ORR activity of Pt_78_Zn_22_/KB and the relative harmlessness of Zn for fuel cells, we carried out the H_2_‐O_2_ PEMFCs test. The Pt_78_Zn_22_/KB with a loading of 0.15 mg cm^–2^ was used as a cathode electrocatalyst. **Figure** **4**a shows the polarization curves of fuel cells with Pt_78_Zn_22_/KB and Pt/C as the cathode. Pt_78_Zn_22_/KB displays an outstanding performance at 0.6 V (1.828 A cm^–2^), which is much better than commercial Pt/C (1.424 A cm^–2^ at 0.6 V). The maximum peak power density of MEA with Pt_78_Zn_22_/KB cathode can reach 1449.48 mW cm^–2^, superior to commercial Pt/C. This excellent performance is one of the best‐reported performance of Pt‐based catalysts (Table S4, Supporting Information). Furthermore, we employed an ADT test protocol suggested by DOE to evaluate the durability of the catalyst, i.e., using square‐wave potential between 0.60 and 0.95 V with a holding time of 3 s at each potential. After the ADTs of 30 000 cycles, compared to commercial Pt/C, Pt_78_Zn_22_/KB exhibited less performance loss. Figure 4b and Figure S20 (Supporting Information) show the fuel cell's polarization curves before and after cycling with Pt_78_Zn_22_/KB and Pt/C as the cathode. After ADT, the peak power density of Pt/C attenuates from 1149 mW cm^–2^ to 735 mW cm^–2^, with a loss of about 36%. In contrast, the peak power density of Pt_78_Zn_22_/KB decreases from 1449 mW cm^–2^ to 1219 mW cm^–2^, with a loss of about 15%, significantly lower than commercial Pt/C. Therefore, the Pt_78_Zn_22_/KB catalyst has better durability as the PEMFC cathode.

In addition to the stability of catalyst itself, the reactive oxygen species (ROS) may also result in the degradation of Nafion membrane and accelerate material corrosion. As reported by previous work, 2,20‐azinobis(3‐ethylbenzthiazoline‐6‐sulfonate) (ABTS) can be oxidized by ROS and result in a change in the absorbance at ≈417 nm in UV/Vis absorption spectroscopy which is mainly manifested as the solution changes from colorless to green (Figure 4c,d).^[^ ^58^ ^]^ As expected, the absorbance of ABTS+H_2_O_2_+Fe/Co/Ni are about 6.8 times, 2.8 times, and 1.3 times that of ABTS+H_2_O_2_+Zn, respectively, which clearly shows that Zn can significantly inhibit the Fenton reaction and the formation of ROS.

**Figure 5:**
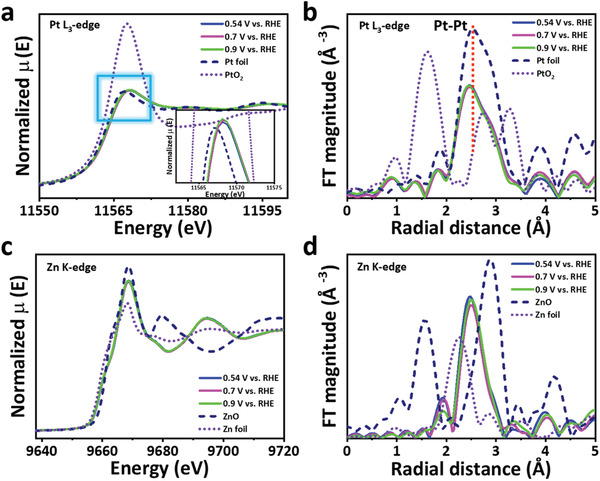
In situ XANES and XAFS analysis. a) In situ Pt L_3_‐edge XANES spectra for Pt_78_Zn_22_/KB NCs with an enlarged view of the area marked by the blue square. b) Pt L_3_‐edge Fourier‐Transform XAFS spectra of Pt_78_Zn_22_/KB with the different potential and reference samples. c) In situ Zn K‐edge XANES spectra for Pt_78_Zn_22_/KB NCs. d) Zn K‐edge Fourier‐Transform XAFS spectra of Pt_78_Zn_22_/KB with the different potential and reference samples.

In situ XAS examinations were carried out to analyze the dynamic changes of the oxidation state and the coordination environment under the ORR‐relevant potentials.^[^ ^59^ ^]^ **Figure** **5**a shows the potential‐dependent Pt L_3_‐edge XANES of Pt_78_Zn_22_/KB, probing the dynamic changes under applied potentials 0.54, 0.7, and 0.9 V (vs RHE). The normalized white line intensity increases slightly with an increasing applied potential (inset in Figure 4a).^[^ ^60^, ^61^ ^]^ Generally speaking, the normalized white line peak intensity is related to the vacancy level in the Pt 5d orbitals near the Fermi level. The increasing vacancies in the 5d orbital may come from the oxygen species' adsorption from the electrolyte following a Temkin isotherm behavior.^[^ ^62^, ^63^ ^]^ But alloying above 0.8 V (vs RHE) will inhibit the chemical adsorption of oxygen species on the Pt surface. The bond length of Pt–Pt (2.44 Å) and Pt–O (1.36 Å) in the Fourier transform (FT) k^3^‐weighted EXAFS spectra of Pt_78_Zn_22_/KB have no significant variation with potential from 0.54 to 0.9 V.^[^ ^63^ ^]^ (Figure 5b). As shown in Figure 5c, Zn K‐edge XANES spectra at different potentials show that the adsorption threshold position of Pt_78_Zn_22_/KB is located between Zn foil and ZnO, indicating that Zn would be slightly oxidized under the condition of applied voltages. However, the white line intensity changes of Zn at the three potentials of 0.54 V/0.7 V/0.9 V are minimal, indicating that Zn has slight corrosion and dissolution. The role of Zn may adjust the electronic structure of Pt and thus improve the ORR performance. Simultaneously, in Figure 5d, the FT k^3^‐weighted EXAFS spectra of Zn K‐edge show that the Zn‐Zn bond length in acid increases with the applied potentials increased, indicating that the strain effect between Pt–Zn increases at high potentials.^[^ ^64^ ^]^

**Figure 6:**
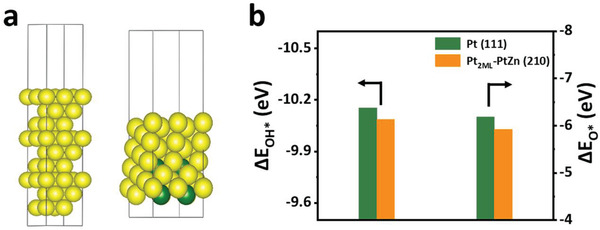
DFT calculations. a) Optimized models of Pt (111) and Pt_2ML_–Pt_78_Zn_22_ (210) in order. Color code: yellow, Pt; green, Zn. b) Δ*E*_OH*_ and Δ*E*_O*_ on Pt (111) and Pt_2ML_–Pt_78_Zn_22_ (210) slabs.

In order to explore the origin of the superior ORR activities of Pt_78_Zn_22_/KB, the density functional theory (DFT) calculations were used to gain an in‐depth understanding of the optimized oxygenate intermediates adsorption energy (*E*_O*_ or *E*_OH*_).^[^ ^65^ ^]^ Taking into account the high‐index facets and the incorporation of Zn atom, the Pt (111) and Pt_2ML_–Pt_78_Zn_22_ (210) with two monolayers “Pt skin” were constructed as calculation models (**Figure** **6**a). The two‐layer “Pt skin” confirmed by STEM‐EDS line‐scanning was set. (Figure 1f). It is generally accepted that the ORR can be inhibited by high oxygen species coverage on Pt, and the adsorption energies of O* and OH* on the surface of an ideal catalyst for ORR are ≈0.2 and ≈0.1 eV weaker than Pt (111), respectively. Generally, the adsorption energy of oxygen species (Δ*E*_O*_ or Δ*E*_OH*_) is a robust descriptor to evaluate ORR performance.^[^ ^66^ ^]^ As shown in Figure 6b, Δ*E*_O*_ or Δ*E*_OH*_ on the Pt surface in the Pt (111) and Pt_2ML_–Pt_78_Zn_22_ (210) models are calculated by DFT. The adsorption energies of O* and OH* on the surface of Pt_2ML_–Pt_78_Zn_22_ (210) are ≈0.21, ≈0.052 eV weaker than Pt (111), which are basically in line with the ideal catalyst standard. The weakened adsorption energy can decrease the affinity of oxygen species generated during oxygen reduction. It has been documented that the downshift of the d‐band center, triggered by lattice strain and ligand effects, can decrease surface reactivity and facilitate the oxygen reduction on Pt.^[^ ^48^, ^67^ ^]^ Based on the above analysis, it can be concluded that incorporating Zn, ultrathin Pt skins, ligand and strain synergistic effects, and the rational Pt/Zn ratio weaken the Pt‐O binding strength, thereby promoting the ORR reaction.

## 3 Conclusion

In conclusion, we prepared concave Pt–Zn nanocubes with high‐index ultrathin Pt skin as efficient and durable electrocatalysts toward ORR. The Pt–Zn nanocubes electrocatalytic studies showed a high mass activity of 1.18 A mg_Pt_^–1^, a specific activity of 3.64 mA cm^–2^ at 0.9 V (vs RHE), and a much lower activity loss than that of commercial Pt/C after 30 000 cycles test. The H_2_–O_2_ fuel cells assembled by Pt_78_Zn_22_/KB catalyst also delivered superior performance to commercial Pt/C. The experiments and theoretical calculations revealed that such excellent performance can be attributed to the incorporation of zinc and the existence of high‐index facets ultrathin Pt‐rich skins, which adjusted the electronic structure of Pt on the surface, and led to improved catalytic activity. This work provides a feasible strategy for designing Pt–M electrocatalysts and helps the energy conversion technology be applied in practice.

## Conflict of Interest

The authors declare no conflict of interest.

## Supporting information

Supporting information

## Data Availability

The data that support the findings of this study are available from the corresponding author upon reasonable request.
