# Supplementary material for: Concave Pt–Zn Nanocubes with High‐Index Faceted Pt Skin as Highly Efficient Oxygen Reduction Catalyst
Source: Adv Sci (Weinh). 2022 Feb 24;9(12):2200147. doi: 10.1002/advs.202200147 (PMC9036018; doi:10.1002/advs.202200147)
Supplement: Supplementary file 1 — Supporting information [file ADVS-9-2200147-s001.pdf]

ADVANCED SCIENCE
Open Access

Supporting Information

for *Adv. Sci.*, DOI 10.1002/advs.202200147

Concave Pt–Zn Nanocubes with High-Index Faceted Pt Skin as Highly Efficient Oxygen Reduction Catalyst

Mengli Liu, Bang-An Lu, Gege Yang, Pengfei Yuan, Huicong Xia, Yajin Wang, Kai Guo, Shuyan Zhao, Jia Liu, Yue Yu, Wenfu Yan, Chung-Li Dong, Jia-Nan Zhang* and Shichun Mu*

WILEY-VCH

Copyright WILEY-VCH Verlag GmbH & Co. KGaA, 69469 Weinheim, Germany, 2016.

Supporting Information

Concave Pt-Zn Nanocubes with High-Index Faceted Pt Skin as Highly Efficient Oxygen Reduction Catalyst

Mengli Liu,[#] Bang-An Lu,[#] Gege Yang, Pengfei Yuan, Huicong Xia, Yajin Wang, Kai Guo, Shuyan Zhao, Jia Liu, Yue Yu, Wenfu Yan, Chung-Li Dong,[*] Jia-Nan Zhang,[*] Shichun Mu

Ms. M. Liu, Dr. B.-A. Lu, Ms. G. Yang, Mr. H. Xia, Ms. Y. Wang, Mr. K. Guo, Ms. S. Zhao, Mr. Y. Yu, Prof. J.-N. Zhang
College of Materials Science and Engineering
Zhengzhou University
Zhengzhou 450000, P. R. China
E-mail: zjn@zzu.edu.cn (J.-N Zhang)

Prof. S. Mu
State Key Laboratory of Advanced Technology for Materials Synthesis and Processing
Wuhan University of Technology
Wuhan 430070, P. R. China

Prof. C.-L. Dong
Department of Physics, Tamkang University, Tamsui 25137, P. R. Taiwan
E-mail: 148558@mail.tku.edu.tw

Dr. P. Yuan
International Joint Research Laboratory for Quantum Functional Materials of Henan Province, and School of Physics and Microelectronics
Zhengzhou University
Zhengzhou 450000, P. R. China

Dr. J. Liu
Shanghai Hydrogen Propulsion Technology Co., Ltd., Shanghai, P.R. China

Prof. W. Yan
State Key Laboratory of Inorganic Synthesis & Preparative Chemistry
Jilin University
Changchun 130000, P. R. China

[#] These authors contributed equally to this work.

Table of Contents

1. **Experimental Details**

2. **Computational Methods and Models**

3. **Supplementary Figures**

4. **Supplementary Tables**

1. Experimental Section

Chemicals and reagents. Platinum (II) acetylacetonate (Pt(acac)$_2$, 98%), Zinc acetylacetonate hydrate (Zn(acac)$_2$, 97%), Cobalt (II) acetylacetonate (Co(acac)$_2$, 99%) and polyvinyl pyrrolidone (PVP) were purchased from Innochem. Acetaldehyde (C_2H_4O, 40 wt.% in H_2O) and isopropyl alcohol (C_3H_8O, analytical reagent, ≥ 99.5%) were obtained from Macklin. Benzyl alcohol ($C_6H_5CH_2OH$, 99%) was bought from Tianjin YongDa Chemical Reagent Co. Ltd. Ethanol absolute (CH_3CH_2OH, analytical reagent, 99%) and acetic acid ($C_2H_4O_2$, analytical reagent, 99%) were acquired from Sinopharm Chemical Reagent Co. Ltd. Perchloric acid ($HClO_4$, analytical reagent, 70%-90%), and Sigma-Aldrich supplied Nafion (5 wt.%). Pt catalyst (20% Pt supported on Vulcan XC-72 carbon) was obtained from Johnson Matthey. All chemicals were purchased commercially and used without further purification. The water (18.2 MΩ/cm) used in all experiments was prepared by an ultra-pure purification system (HHitech).

***Synthesis of Pt-Zn Nanocrystals**:* In a typical synthesis, 16 mg of Pt(acac)$_2$, 20 mg of Zn(acac)$_2$, and 240 mg of polyvinyl pyrrolidone (PVP) were added into a mixed solution containing 6 mL of benzyl alcohol and 6 mL of acetaldehyde. The mixture solution was stirred vigorously for 1 h to form a homogeneous solution and then transferred into a 25mL Teflon-lined autoclave. Then, the vessel was sealed and heated from room temperature to 180°C in 30 min and kept at this temperature for another 8 h before it was naturally cooled to room temperature. The resulting products were collected by centrifugation (12,000 rpm, 10 min) and washed three times with cyclohexane and ethanol (1:1). The obtained products were heated and acid-washed with acetic acid, collected by centrifugation, and washed with ethanol several times, then dried at 60°C for the night. The obtained Pt-Zn nanocrystals

loaded on Ketjen Black (KB) to get Pt-Zn/KB. A series of contrast samples without Zn(acac)$_2$ and different proportions of Pt/Zn were prepared. The ratios of Pt/Zn determined by the inductively coupled plasma-optical emission spectrometer (ICP-OES) were 46.3:53.7, 78.2:21.8 and 100:0, labeled as $Pt_{46}Zn_{54}$/KB, $Pt_{78}Zn_{22}$/KB and pure Pt/KB, respectively. As listed in Table S1, the weight percent of Pt and Zn are analyzed by ICP-OES analysis.

Characterizations. The morphology of the samples was characterized by transmission electron microscopes (TEM, FEI Tecnai G220) with an accelerating voltage of 200 kV and field-emission scanning electron microscope (FE-SEM, JEORJSM-6700F). JEOL JEM-ARM200F obtained the HAADF-STEM images at an accelerating voltage of 200 kV. The crystal phases present in each sample were identified using powder X-ray diffraction (XRD) patterns were recorded on a Y-2000 X-ray Diffractometer with copper Kα radiation (λ = 1.5406 Å) at 40 kV, 40 mA. The X-ray photoelectron spectroscopy (XPS) measurements were performed with an ESCA LAB 250 spectrometer on a focused monochromatic Al Kα line (1486.6 eV) X-ray beam with a diameter of 200 μm. The Raman measurements were taken on a Renishaw spectrometer at 532 nm on a Renishaw Microscope System RM2000. The Pt loadings were determined by the inductively coupled plasma-optical emission spectrometer (ICP-OES. SHIMADZU ICPE-9820) in this work.

In situ X-ray Absorption Fine Structure (XAFS). The as-prepared electrocatalyst was coated on RDEs. Then the pretreatment was as follows: the working electrodes were pretreated by voltammetric cycles between 0.2 and 0.8 V at a rate of 50 mV s^{-1} in O_2-saturated 0.1 M $HClO_4$ aqueous solution. Subsequently, in situ/operando XAFS spectra were collected. At each set potential (0.54 V, 0.7 V, and 0.9 V), the in situ X-ray absorption signals were collected when the working electrode reached a steady

state. The potential-dependent in situ Pt L_3-edge X-ray absorption near edge structure (XANES) and the extended X-ray absorption fine structure (EXAFS) were investigated at the Taiwan Photon Source 44A beamline and BW14 of SSRF. References, such as Pt and Zn foils, are used to calibrate the beamline energy and compare samples. Fluorescence mode detection was performed using a Si (111) double-crystal monochromator. An ion chamber detector for samples and the total electron yield was used to measure samples with high concentrations, such as references. The EXAFS raw data were then background-subtracted, normalized, and Fourier transformed by the standard procedures with the IFEFFIT package.

Electrocatalytic measurements. Electrochemical experiments were conducted on a CHI760E electrochemical workstation (CH Instrument Co., USA). CV, RDE, and RRDE measurements (Pine Research Instrument, USA) were performed using a standard three-electrode system. For the preparation of the working electrode, 2 mg of $Pt_{78}Zn_{22}$/KB, $Pt_{46}Zn_{54}$/KB, pure Pt/KB, and commercial Pt/C catalysts were dispersed in 1 mL mixture of ethanol and 5% Nafion solution (v:v = 49:1) and sonicated for 1 h to form homogeneous catalyst ink. To obtain a uniform thin catalyst film on the electrode, the desired aliquot was dropped onto a glassy carbon rotating disk electrode (RDE, ϕ = 5 mm) and dried in the air at room temperature，

For the RRDE measurements, the disk electrode was scanned cathodically at a rate of 10 mV $s^{-1,}$ and the ring potential was kept at 1.5 V versus RHE. The peroxide percentage and the electron transfer number (n) were determined by the following equations (ref):

$$HO_2^- = 200 \times \frac{I_R/N}{I_D + I_R/N} \qquad (4)$$

$$n = 4 \times \frac{I_D}{I_D + I_R/N} \qquad (5)$$

where I_D is disk current, I_R is ring current, and N is the current collection efficiency of the Pt ring. N was determined to be 0.40.

MEA Preparation and Fuel Cell Testing. Catalysts were mixed with Nafion, isopropyl alcohol, and water by ultrasonication for 1 hour to form homogeneous ink. The ink has then sprayed the ink onto Nafion 211 membrane at 70°C. Fuel cell testing was performed in a single cell using a commercial fuel cell test system (Scribner 850e, Hephas Energy Corporation). The MEA was sandwiched between two graphite plates with single serpentine flow channels. The cell was operated at 80°C with a back pressure of 150 kPa. Pure hydrogen and air/oxygen, with 100% relative humidity (RH), were supplied to the anode and cathode at a gas flow rate of 500-1000 sccm. Fuel cell polarization curves were recorded using potential step mode with 50 mV/point (holding 2 min for each point).

Fenton-like reactivity measurements: The production of intermediate hydrogen peroxide was evaluated via a reported method.[1] Briefly, the metal ions (Zn^{2+}, Ni^{2+}, Co^{2+}, and Fe^{3+}) were ultrasonically dissolved in 0.1 M $HClO_4$ solution respectively to achieve a 40 μg mL^{-1} homogeneous suspension. The desired amounts of 2,20-azinobis(3-ethylbenzthiazoline-6-sulfonate) (ABTS), and hydrogen peroxide were sequentially added to reach the concentrations of 2 mM and 20 mM, respectively. After 5 min, the solution was diluted with 0.1 M $HClO_4$ (3:100) and characterized by UV-Vis spectroscopy (SHIMADZU UV-1900 Series UV-Vis Spectrophotometer).

2. Computational methods and models. First-Principles calculations were carried out within the density functional theory framework.[2] The projector-augmented wave (PAW) method[3, 4] and the generalized gradient approximation (GGA)[5] for the exchange-correlation energy functional, as implemented in the Vienna ab initio simulation package (VASP)[6-8] were used. The GGA calculation

was performed with the Perdew-Burke-Ernzerhof (PBE)[9] exchange-correlation potential. Plane-wave cutoff energy of 400 eV was used.

Based on the experimental results, two different supports were considered: pure Pt (111), Pt-Zn (210) with high-index facets with ultra-thin Pt skin adsorbed on the Pt-Zn (210) surface. For Pt (111), nine layers with the bottom three fixed was used in all the calculations. A 5x5 k-points was used for the optimization of Pt (111). For Pt-Zn (210), twelve layers of Pt-Zn (210) with the bottom three fixed were used. A 3x3 k-points was used for the optimization of Pt-Zn (210). All the other atoms were fully relaxed with tolerance in total energy of 0.1 m eV, and the forces on each atom were less than 0.02 eV/Å.

Because of the decompose of O_2 on Pt， the process of the ORR is the following four-electron steps:

Dissociative:

$$O_2 + 2^{*} \Rightarrow 2O^{*} \qquad (6)$$

$$2O^{*} + 2H^{+} + 2e^{-} \Rightarrow 2OH^{*} \qquad (7)$$

$$2OH^{*} + 2H^{+} + 2e^{-} \Rightarrow 2H_2O + 2^{*} \qquad (8)$$

Associative:

$$O_2 + * \Rightarrow O_2^{*} \qquad (9)$$

$$O_2^{*} + H^{+} + e^{-} \Rightarrow OOH^{*} \qquad (10)$$

$$OOH^{*} + H^{+} + e^{-} \Rightarrow O^{*} + H_2O \qquad (11)$$

$$O^{*} + H^{+} + e^{-} \Rightarrow OH^{*} \qquad (12)$$

$$OH^* + H^+ + e^- \Rightarrow H_2O \quad (13)$$

where * represents an active site on the corresponding surface.

The adsorption energy (ΔE_{ads}) for ORR was calculated as:

$$E_{\mathrm{ads}} = E_{\mathrm{substrate+adsorbate}} - E_{\mathrm{substrate}} - E_{\mathrm{adsorbate}} \quad (14)$$

The adsorption free energy (ΔG_{ads}) is obtained by

$$\Delta G_{\mathrm{ads}} = \Delta E + \Delta ZPE - T\Delta S + \Delta G_{\mathrm{U}} + \Delta G_{\mathrm{pH}} \quad (15)$$

Where ΔE is the change of the total reaction energy obtained from DFT calculation, ΔZPE and ΔS are the contributions to the free energy from the zero-point vibration energy and entropy, respectively. T is the temperature (300K), and ΔS is the change of the entropy. ΔG_U = -eU, where U is the potential at the electrode and e is the transferred charge. ΔG_{pH} = kB•T×ln10×pH where kB is the Boltzmann constant and T = 300 K. ΔG_{pH} is the correction of the H^+ free energy. The concentration dependence of the entropy has corrected the free energy of H^+ ions:

$$G(\mathrm{pH}) = -kT \ln[\mathrm{H}^+] = kT \ln 10^* \mathrm{pH} \quad (16)$$

(0.059526 for 0.1 M $HClO_4$).

3. Supplementary Figures

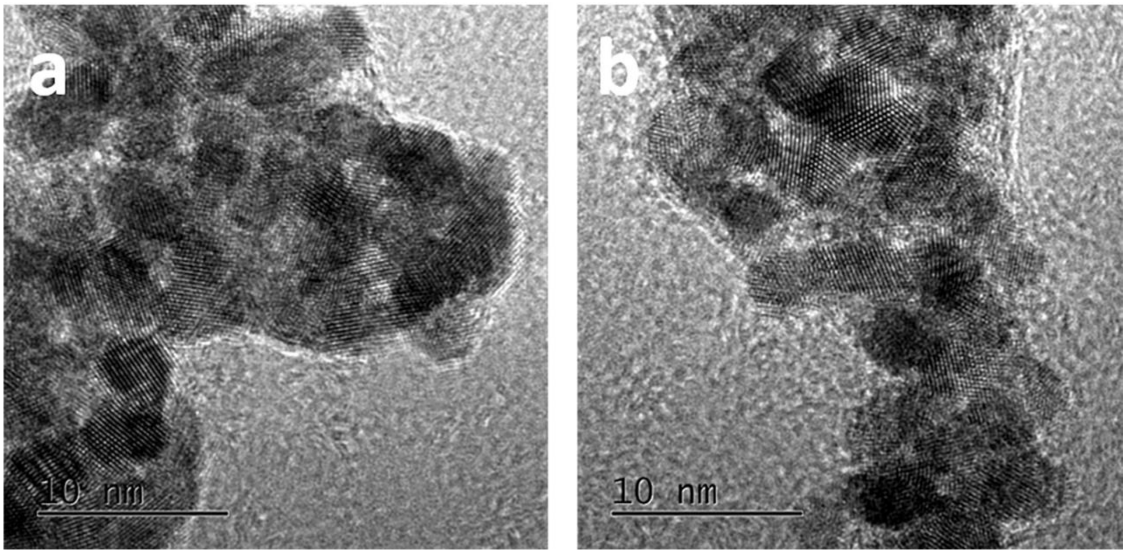

Figure S1. a) and b) TEM images of $Pt_{78}Zn_{22}$ nanocrystals without the addition of acetaldehyde.

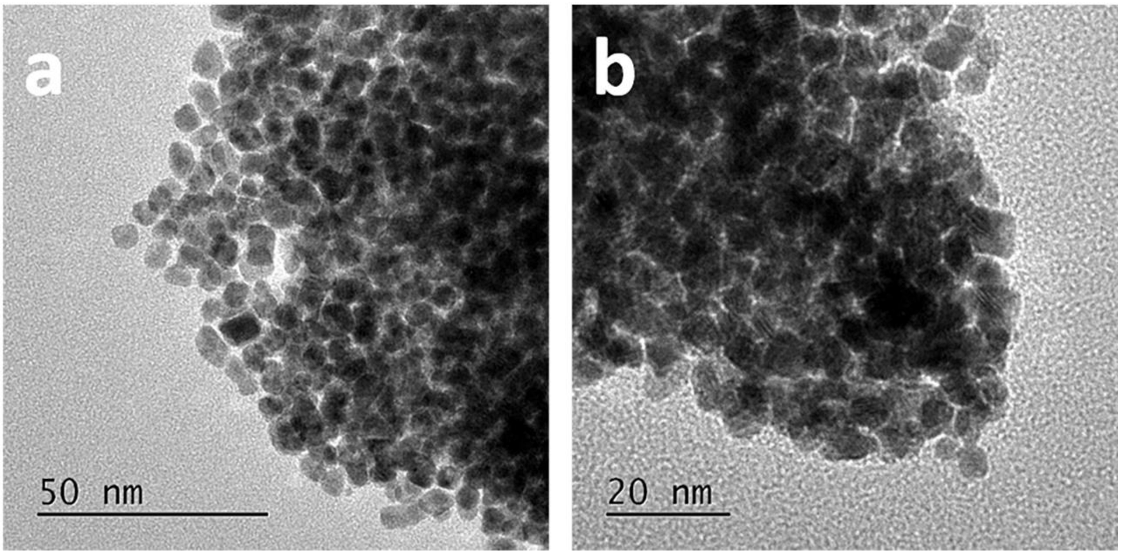

Figure S2. a) and b) TEM images of $Pt_{78}Zn_{22}$ nanocrystals with 2 mL acetaldehyde.

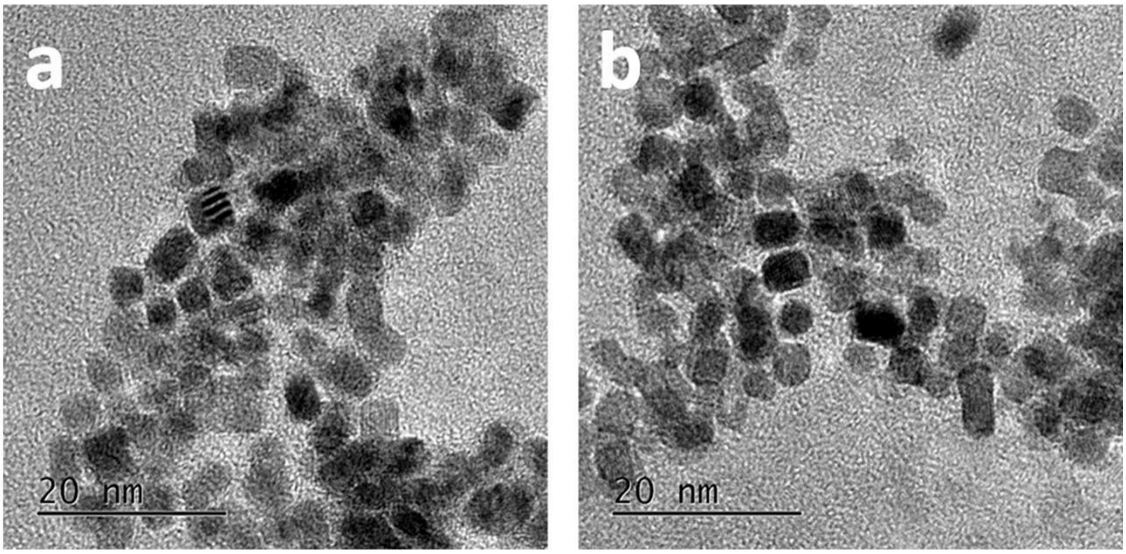

Figure S3. a) and b) TEM images of $Pt_{78}Zn_{22}$ nanocrystals with 4 mL acetaldehyde.

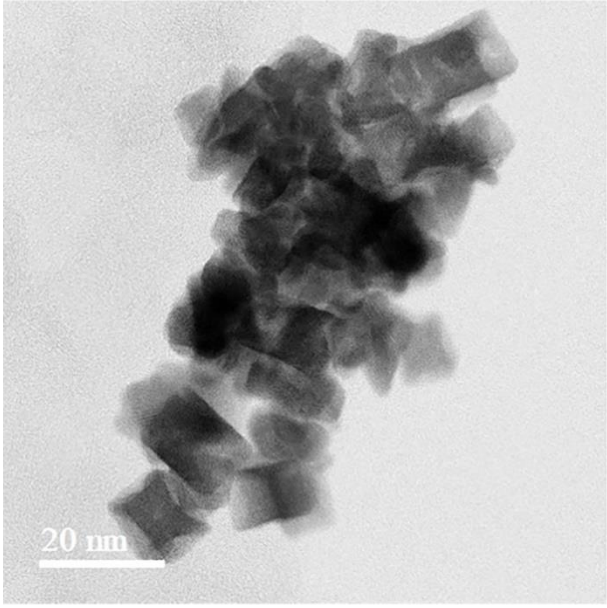

Figure S4. HAADF-STEM image of $Pt_{78}Zn_{22}$ nanocubes with 6 mL acetaldehyde.

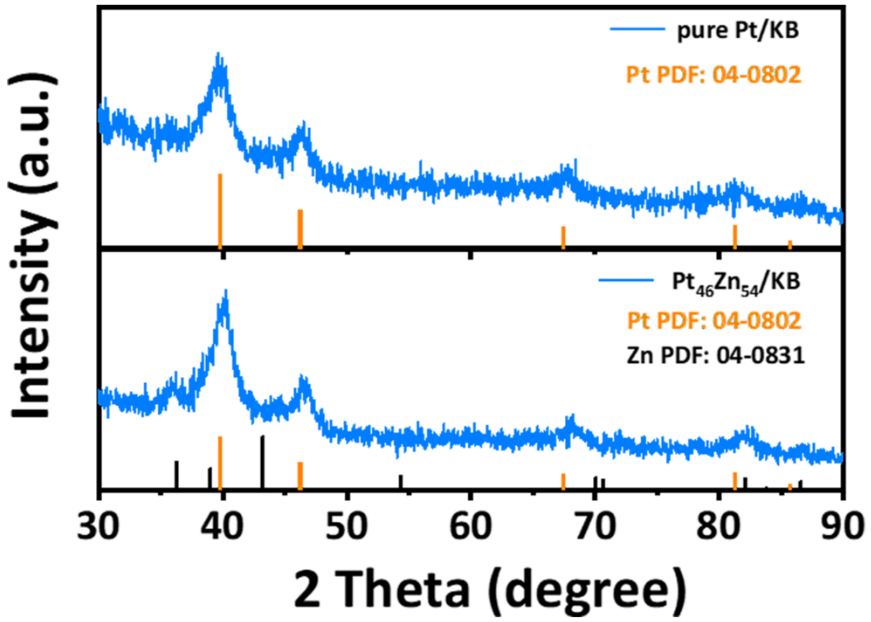

Figure S5. XRD patterns of pure Pt/KB and $Pt_{46}Zn_{54}$/KB.

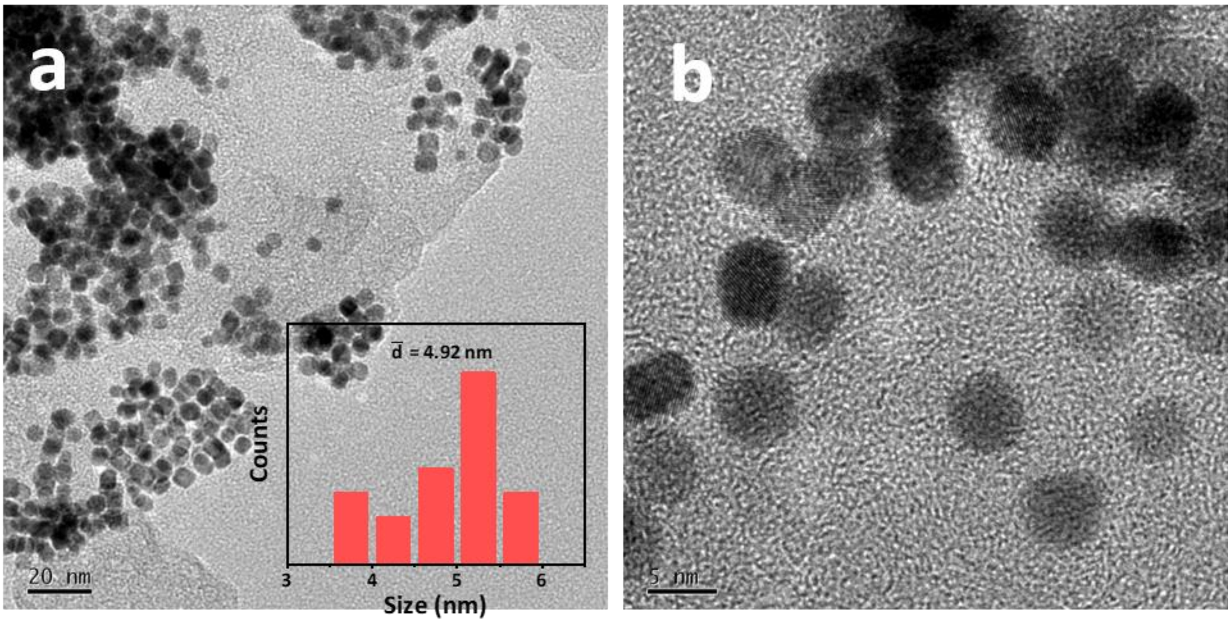

Figure S6. TEM images of pure Pt/KB at (a) low and (b) high magnifications.

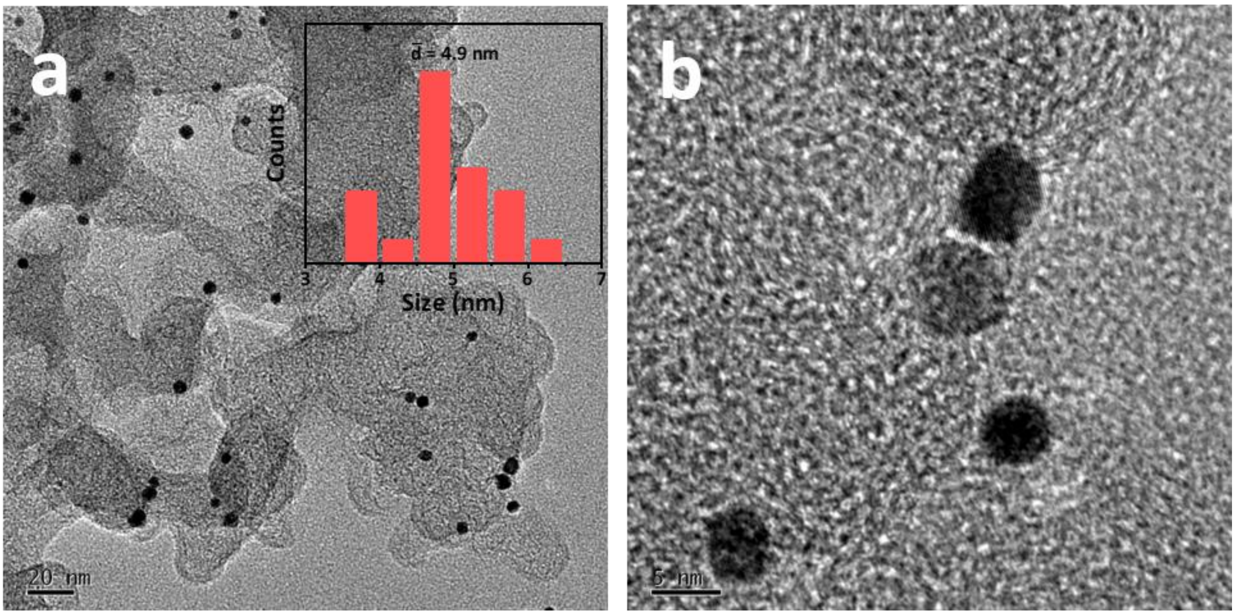

Figure S7. TEM images of $Pt_{46}Zn_{54}$/KB at a) low and b) high magnifications.

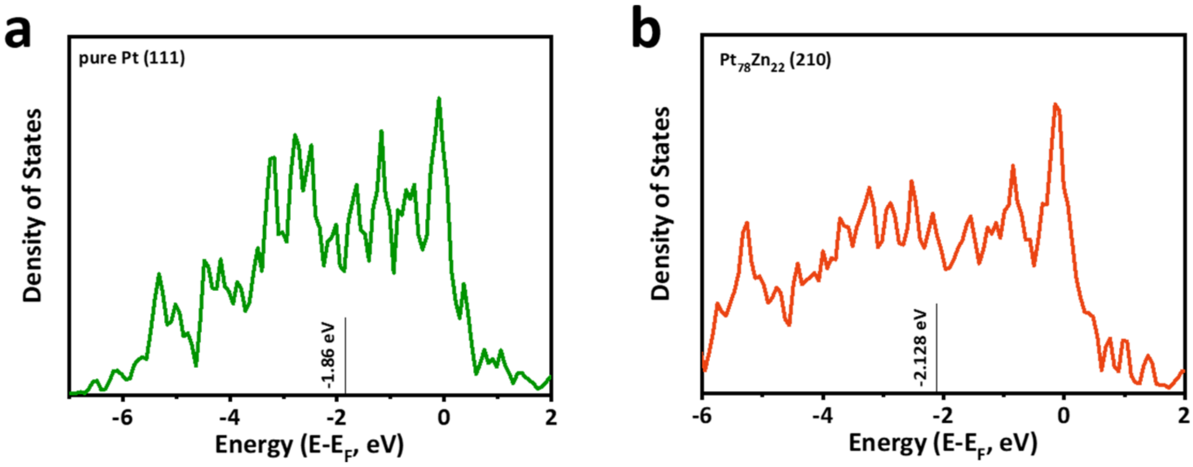

Figure S8. Projected electronic density of states of surface atoms on pure Pt (111) and $Pt_{78}Zn_{22}$ (210).

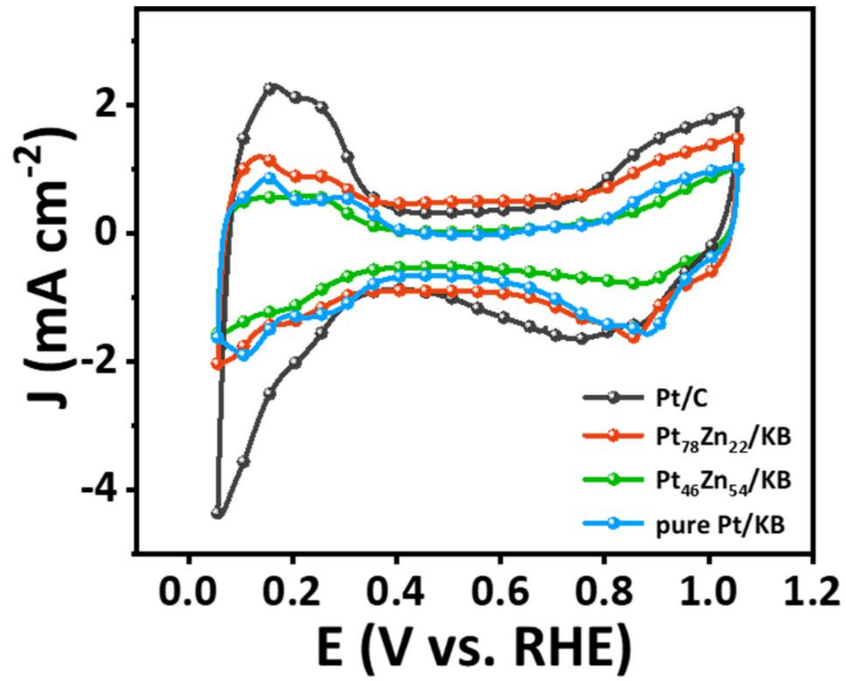

Figure S9. CV curves of pure Pt/KB, $Pt_{46}Zn_{54}$/KB, $Pt_{78}Zn_{22}$/KB, commercial Pt/C in Ar-saturated 0.1 M $HClO_4$.

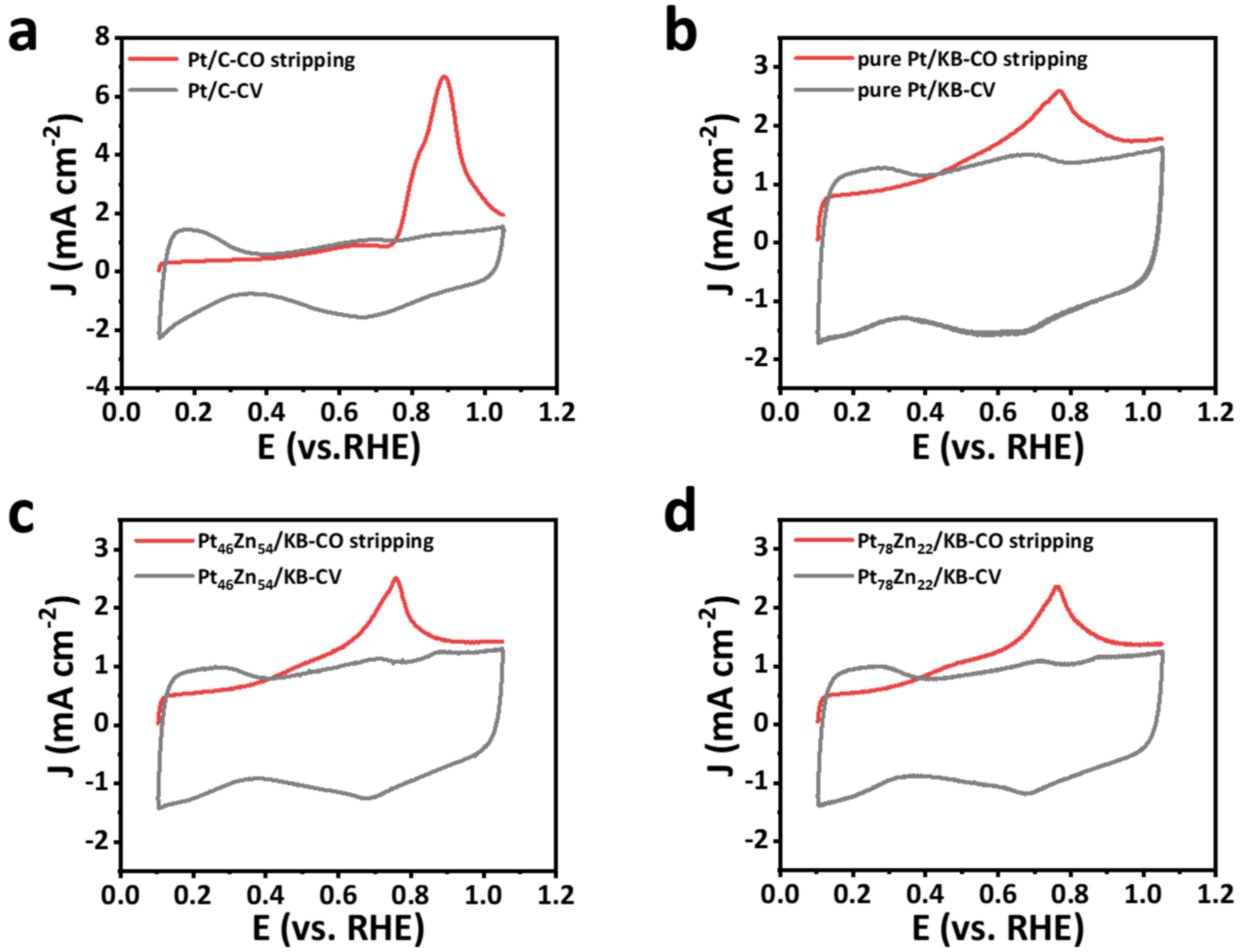

Figure S10. CO stripping curves of commercial Pt/C, pure Pt/KB, $Pt_{46}Zn_{54}$/KB and $Pt_{78}Zn_{22}$/KB.

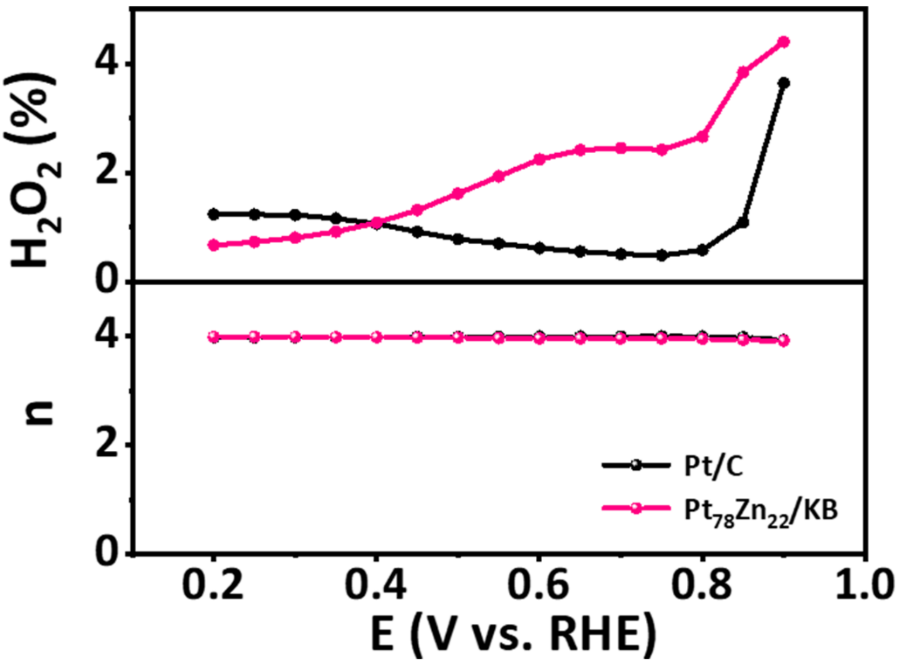

Figure S11. H_2O_2 yield and electron transfer number of commercial Pt/C and $Pt_{78}Zn_{22}$/KB.

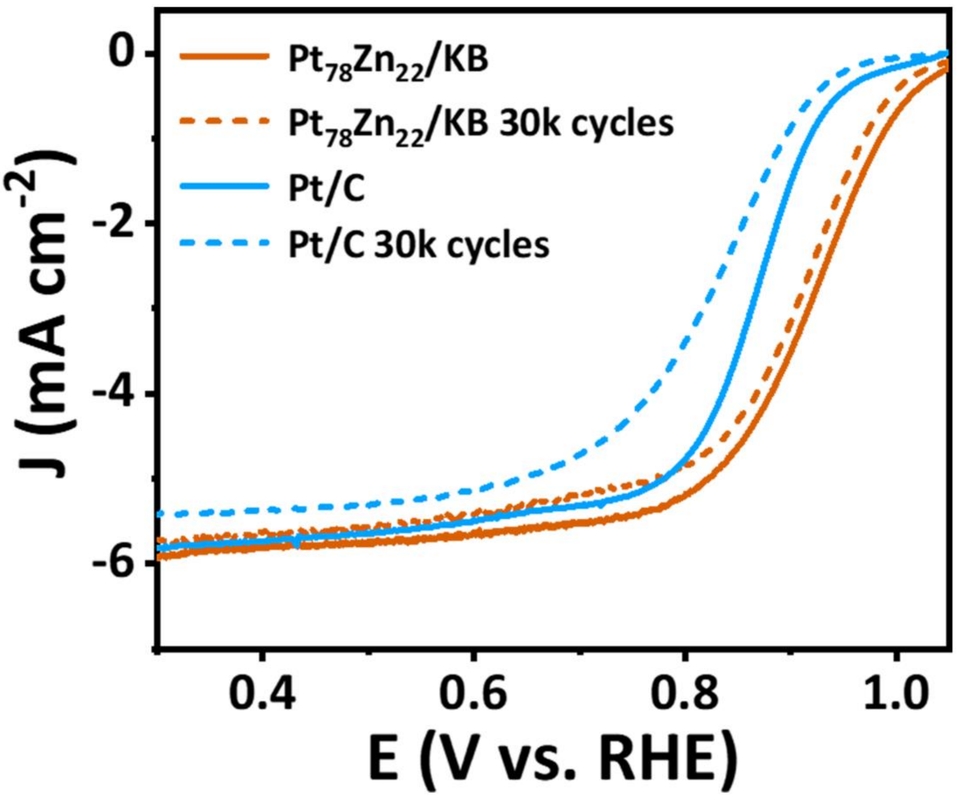

Figure S12. ORR polarization LSV curves of $Pt_{78}Zn_{22}$/KB and Pt/C measurement before and after 30k potential cycles at the scan rate of 50 mV s^{-1} with the rotation speed of 1600 rpm.

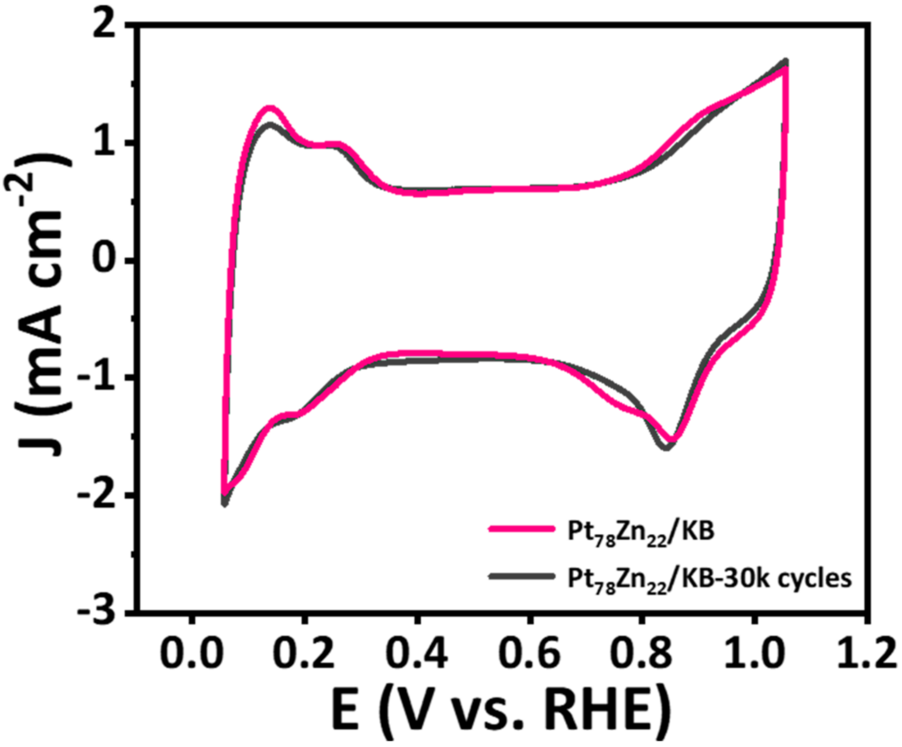

Figure S13. The CV curves of $Pt_{78}Zn_{22}$/KB before and after 30k potential cycles.

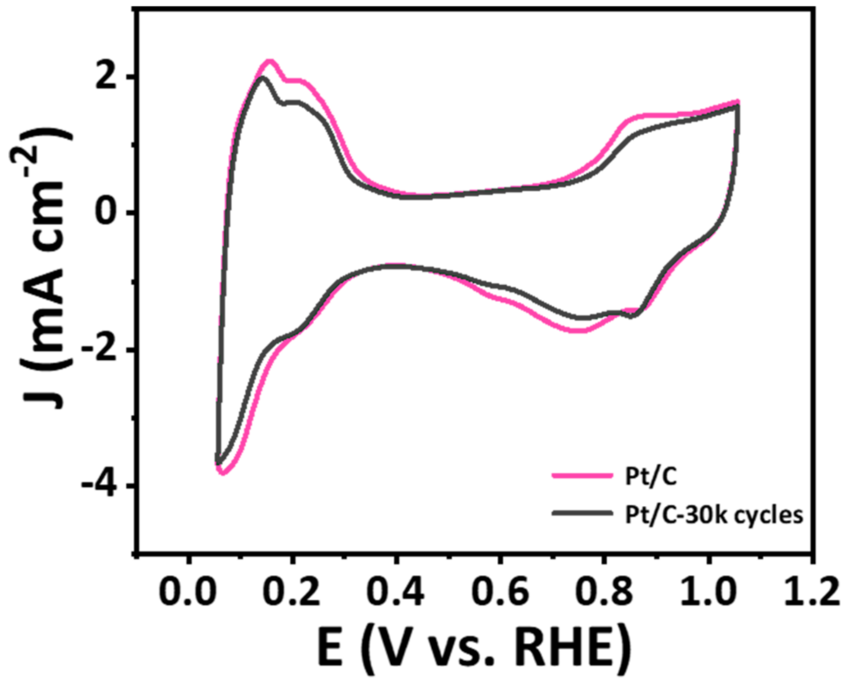

Figure S14. The CV curves for commercial Pt/C before and after the 30k potential cycles.

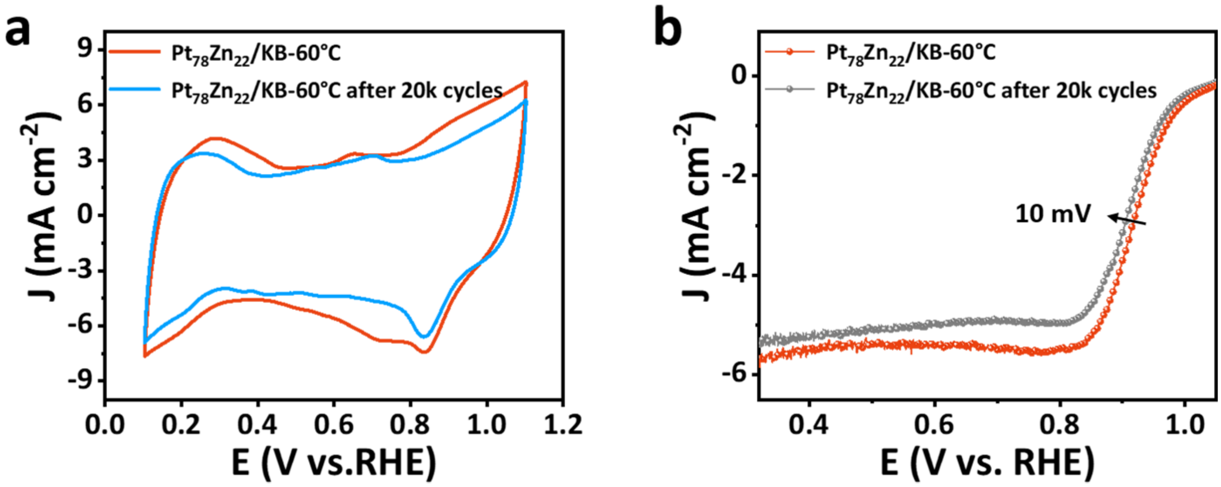

Figure S15. a) CV curves of $Pt_{78}Zn_{22}$/KB in Ar-saturated 0.1 M $HClO_4$ before and after ADTs at 60 ℃. b) ORR polarization curves of $Pt_{78}Zn_{22}$/KB in O_2-saturated 0.1 M $HClO_4$ before and after ADTs at 60 ℃.

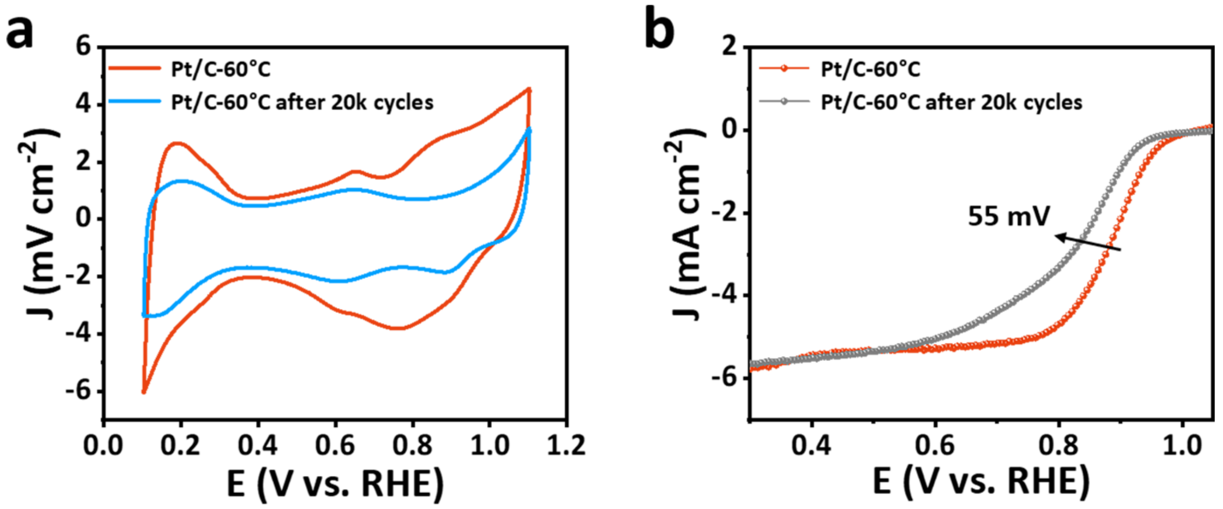

Figure S16. a) CV curves of commercial Pt/C in Ar-saturated 0.1 M $HClO_4$ before and after ADTs at 60 °C. b) ORR polarization curves of commercial Pt/C in O_2-saturated 0.1 M $HClO_4$ before and after ADTs at 60 °C.

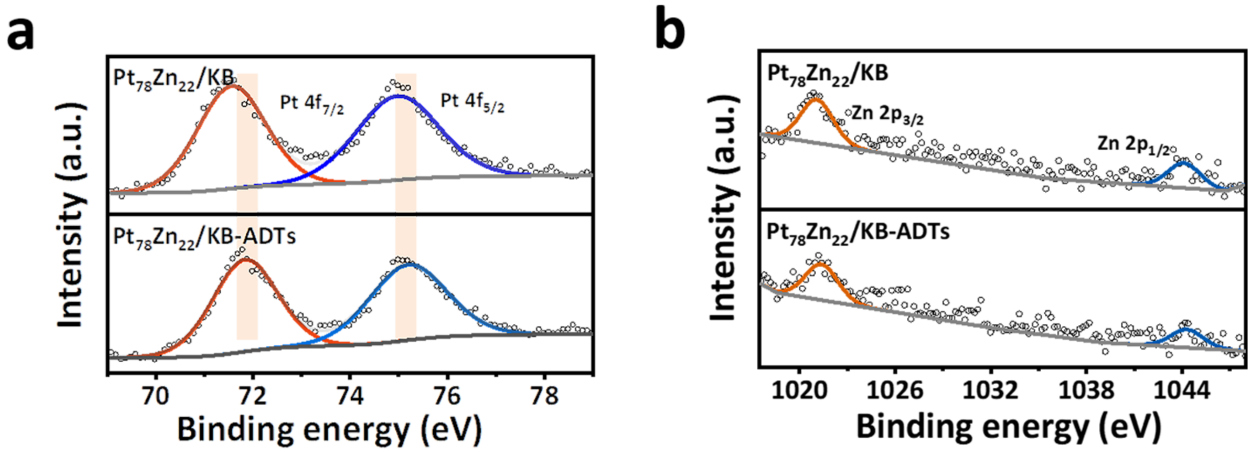

Figure S17. a) Pt 4f XPS spectra of $Pt_{78}Zn_{22}$/KB before and after ADTs. b) Zn 2p XPS spectra of $Pt_{78}Zn_{22}$/KB before and after ADTs.

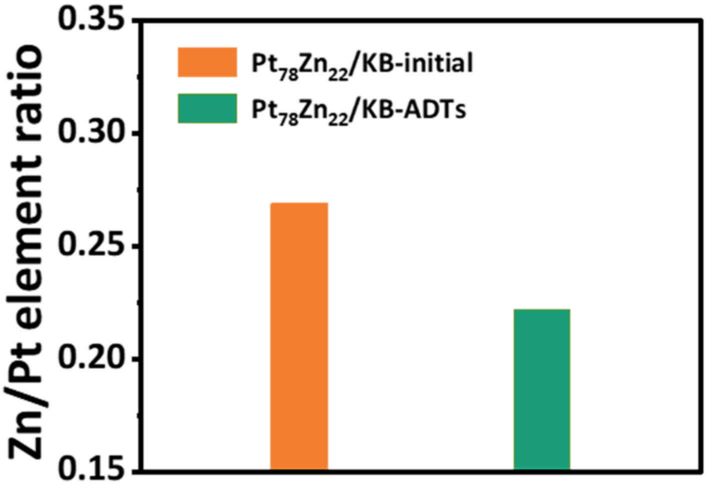

Figure S18. The element ratio of Zn/Pt was calculated by XPS before and after the ADTs.

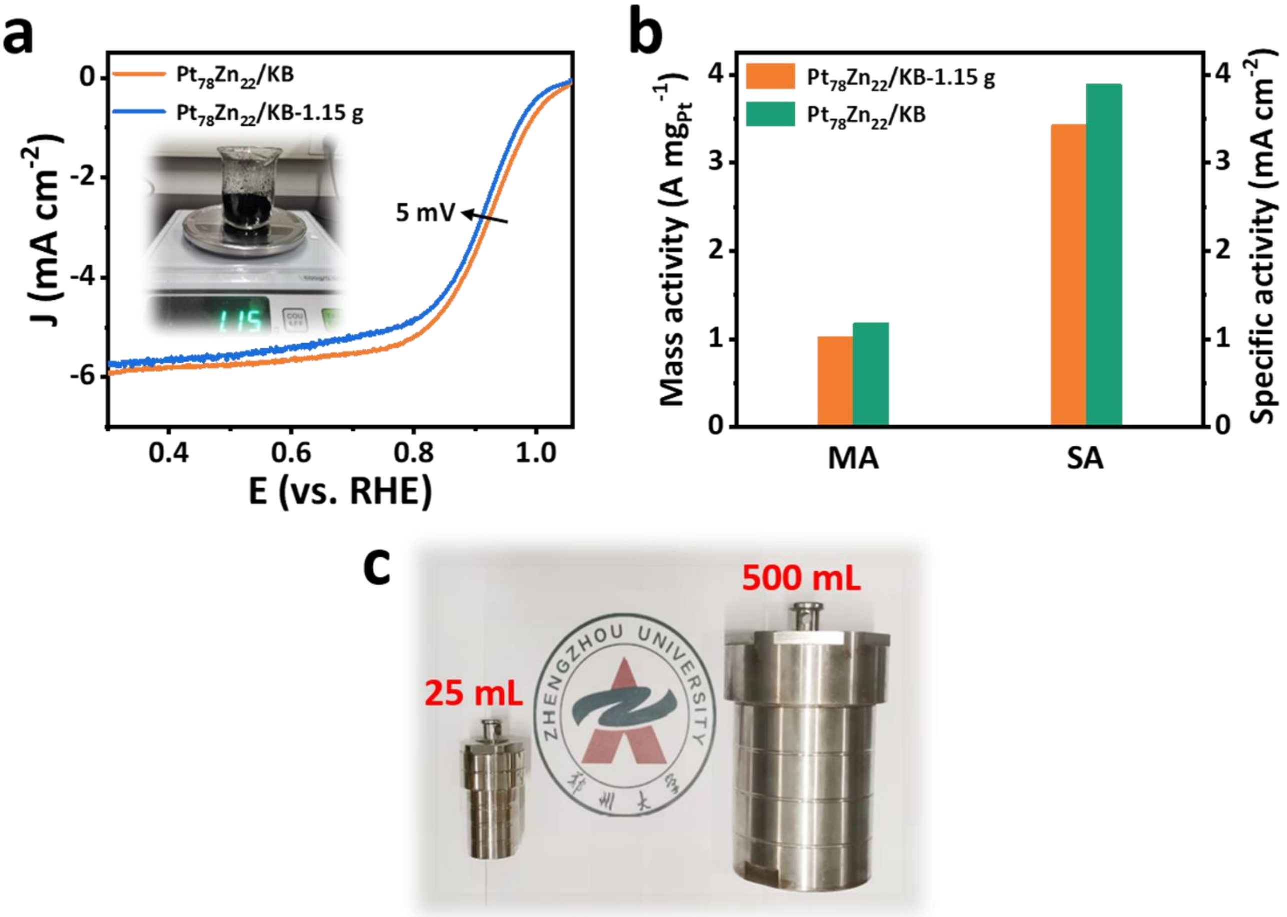

Figure S19. a) Polarization curves of $Pt_{78}Zn_{22}$/KB and $Pt_{78}Zn_{22}$/KB-1.15 g in O_2-saturated 0.1 M $HClO_4$ at a rotation rate of 1600 rpm (scan rate = 5 mV s^{-1}). Inset: photograph of 1.15 g $Pt_{78}Zn_{22}$/KB sample. b) Mass and specific activities of $Pt_{78}Zn_{22}$/KB and $Pt_{78}Zn_{22}$/KB-1.15 g at 0.9V. c) Photograph of 25 mL (left) and 500 mL (right) Teflon-lined autoclaves.

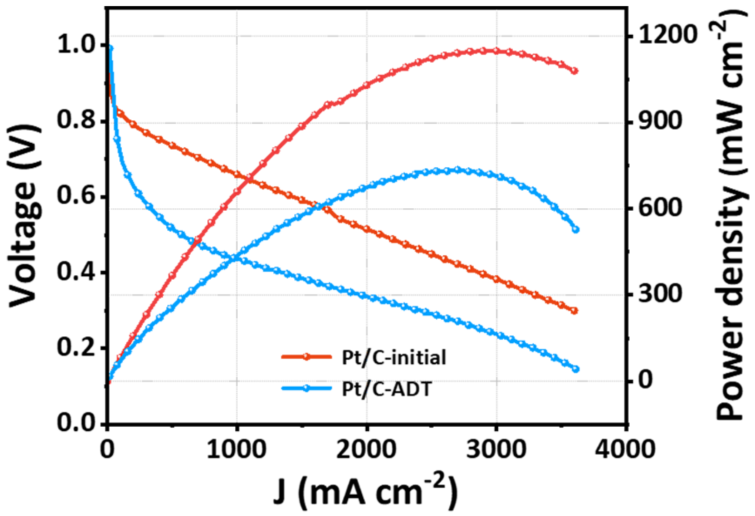

Figure S20. H_2-O_2 fuel cell polarization plots before and after ADTs with Pt/C as the cathode catalyst.

4. Supplementary Tables

Table S1. The weight percent of Pt and Zn in $Pt_{78}Zn_{22}$/KB, $Pt_{46}Zn_{54}$/KB, pure Pt/KB catalysts were shown clearly by ICP-OES.

Catalysts	Pt (wt.%)	Zn (wt.%)
$Pt_{78}Zn_{22}$/KB	24.02	5.71
$Pt_{46}Zn_{54}$/KB	13.10	13.98
Pure Pt/KB	25.75	--

Table S2. Projection angles and geometrical parameters of concave nanocubes bounded by different types of high-index facets.

**{*hk*0}**	**α/°**
{310}	18.4
{310}	20.6
{310}	23.3
{310}	15.9

Table S3. Comparison of the ECSA of $Pt_{78}Zn_{22}$/KB, $Pt_{46}Zn_{54}$/KB, pure Pt/KB and commercial Pt/C determined by CO-stripping and H_{upd}.

Catalysts	CO-stripping ($m^2\ g_{Pt}^{-1}$)	H_{upd} ($m^2\ g_{Pt}^{-1}$)
$Pt_{78}Zn_{22}$/KB	32.38	29.15
$Pt_{46}Zn_{54}$/KB	35.14	26.4
Pure Pt/KB	31.92	27.37
Commercial Pt/C	63.8	62.8

Table S4. Comparisons of the ORR electrocatalytic activity in recently published papers.

Catalysts	MA ($A\ mg_{Pt}^{-1}$)	SA ($mA\ cm^{-2}$)	ECSA ($m^2\ g^{-1}$)	Fuel cells	
				Current density ($A\ cm^{-2}$ at 0.6 V)	Peak power density ($W\ cm^{-2}$)
**$Pt_{78}Zn_{22}$/KB NCs (This work)**	**1.18**	**3.64**	**32.38**	**1.828**	**1.449**
PtFe/rGO[10]	1.96	4.1	42	/	/
A-MS-$Pt_{1.5}Ni$[11]	1.89	7.7	24.9	/	/
$L1_0$-PtZn-C[12]	1.02	1.68	61.1	0.549 (0.8V)	2.00
PtNi BNCs/C[13]	3.52	5.16	68.2	1.5	0.92
PtGa NWs/C[14]	1.85	/	53	/	0.9
PtCu/C[15]	0.1	0.24	/	/	/
Ga-PtNi/C[16]	1.24	2.53	49	0.7	0.42
Pt-Pd[17]	1.23	0.99	124.1	/	/
Rh-Pt NWs[18]	1.41	1.63	86.4	/	/
Pt_3Co NWs[19]	0.31	1.04	/	1.5	1.22
$Pt_{75}Co_{25}$ alloy[20]	0.97	2.06	50.16	/	/

References

[1] E. Luo, H. Zhang, X. Wang, L. Gao, L. Gong, T. Zhao, Z. Jin, J. Ge, Z. Jiang, C. Liu, W. Xing, *Angew. Chem. Int. Ed.* **2019**, *58*, 12469.

[2] W. Kohn, L. J. Sham, *Phys. Rev.* **1965**, *140*, A1133.

[3] G. Kresse, D. Joubert, *Phys. Rev. B* **1999**, *59*, 1758.

[4] P. E. Blochl, *Phys. Rev. B* **1994**, *50*, 17953.

[5] J. P. Perdew, Y. Wang, *Phys. Rev. B* **1992**, *45*, 13244.

[6] G. Kresse, J. Hafner, *Phys. Rev. B* **1993**, *47*, 558.

[7] G. Kresse, J. Furthmuller, *Phys. Rev. B* **1996**, *54*, 11169.

[8] G. Kresse, J. Furthmüller, *Comput. Mater. Sci.* **1996**, *6*, 15.

[9] J. P. Perdew, K. Burke, M. Ernzerhof, *Phys. Rev. Lett.* **1996**, *77*, 3865.

[10] T. Y. Yoo, J. M. Yoo, A. K. Sinha, M. S. Bootharaju, E. Jung, H. S. Lee, B. H. Lee, J. Kim, W. H. Antink, Y. M. Kim, J. Lee, E. Lee, D. W. Lee, S. P. Cho, S. J. Yoo, Y. E. Sung, T. Hyeon, *J. Am. Chem. Soc.* **2020**, *142*, 14190.

[11] F. Kong, Z. Ren, M. Norouzi Banis, L. Du, X. Zhou, G. Chen, L. Zhang, J. Li, S. Wang, M. Li, K. Doyle-Davis, Y. Ma, R. Li, A. Young, L. Yang, M. Markiewicz, Y. Tong, G. Yin, C. Du, J. Luo, X. Sun, *ACS Catal.* **2020**, *10*, 4205.

[12] J. Liang, Z. Zhao, N. Li, X. Wang, S. Li, X. Liu, T. Wang, G. Lu, D. Wang, B. J. Hwang, Y. Huang, D. Su, Q. Li, *Adv. Energy Mater.* **2020**, *10*, 2000179.

[13] X. Tian, X. Zhao, Y. Q. Su, L. Wang, H. Wang, D. Dang, B. Chi, H. Liu, E. J. M. Hensen, X. W. D. Lou, B. Y. Xia, *Science* **2019**, *366*, 850.

[14] L. Gao, X. Li, Z. Yao, H. Bai, Y. Lu, C. Ma, S. Lu, Z. Peng, J. Yang, A. Pan, H. Huang, *J. Am. Chem. Soc.* **2019**, *141*, 18083.

[15] J. T. L. Gamler, A. Leonardi, H. M. Ashberry, N. N. Daanen, Y. Losovyj, R. R. Unocic, M. Engel, S. E. Skrabalak, *ACS Nano* **2019**, *13*, 4008.

[16] J. Lim, H. Shin, M. Kim, H. Lee, K. S. Lee, Y. Kwon, D. Song, S. Oh, H. Kim, E. Cho, *Nano Lett.* **2018**, *18*, 2450.

[17] M. Liu, Z. Lyu, Y. Zhang, R. Chen, M. Xie, Y. Xia, *Nano Lett.* **2021**, *21*, 2248.

[18] H. Huang, K. Li, Z. Chen, L. Luo, Y. Gu, D. Zhang, C. Ma, R. Si, J. Yang, Z. Peng, J. Zeng, *J. Am. Chem. Soc.* **2017**, *139*, 8152.

[19] H. Y. Kim, J. M. Kim, Y. Ha, J. Woo, A. Byun, T. J. Shin, K. H. Park, H. Y. Jeong, H. Kim, J. Y. Kim, S. H. Joo, *ACS Catal.* **2019**, *9*, 11242.

[20] D. S. Choi, A. W. Robertson, J. H. Warner, S. O. Kim, H. Kim, *Adv. Mater.* **2016**, *28*, 7115.
